# Cellular Stress Pathways Are Linked to Acetamiprid-Induced Apoptosis in SH-SY5Y Neural Cells

**DOI:** 10.3390/biology10090820

**Published:** 2021-08-24

**Authors:** Ezgi Öztaş, Mehtap Kara, Tuğçe Boran, Enes Bişirir, Ecem Fatma Karaman, Engin Kaptan, Gül Özhan

**Affiliations:** 1 Department of Pharmaceutical Toxicology, Faculty of Pharmacy, Istanbul University, Istanbul 34116, Turkey; mehtap.kara@istanbul.edu.tr (M.K.); tugce.boran.1@istanbul.edu.tr (T.B.); enes.bisirir@sbu.edu.tr (E.B.); ekaraman@biruni.edu.tr (E.F.K.); gulozhan@istanbul.edu.tr (G.Ö.); 2 Department of Pharmaceutical Toxicology, Faculty of Pharmacy, University of Health Sciences, Istanbul 34668, Turkey; 3 Department of Pharmaceutical Toxicology, Faculty of Pharmacy, Biruni University, Istanbul 34010, Turkey; 4 Department of Biology, Faculty of Sciences, Istanbul University, Istanbul 34134, Turkey; engkaptan@istanbul.edu.tr

**Keywords:** acetamiprid, oxidative stress, endoplasmic reticulum stress, apoptosis/necrosis, human neuroblastoma cells

## Abstract

**Simple Summary:**

Neonicotinoids constitute more than one-quarter of the insecticides on the market. Acetamiprid, a widely used neonicotinoid, has been found to be linked with neurological symptoms and there is an urge to understand its molecular mechanisms. It decreased cellular viability in millimole concentrations after 24 h in SH-SY5Y neural cells. Additionally, it increased reactive oxygen species, intracellular calcium and endoplasmic reticulum stress. Since overwhelmed cellular stress can destroy cellular structures and cause cell death, we also evaluated cellular death mechanisms. Acetamiprid induced apoptosis rather than necrosis indicating that cells undergo suicide initiated by self-generated death signals. Even though acetamiprid is considered to be a safe option in the struggle against harmful agricultural insects, these results suggest that the widespread use should be taken under strict control in order not to cause damage to the mammals.

**Abstract:**

Acetamiprid (ACE), a commonly used neonicotinoid insecticide, is correlated with neurological symptoms, immunotoxicity and hepatotoxicity. Cellular stress and damage could play an important role in ACE-induced neurotoxicity; however, its mechanism has not been fully understood. We evaluated the effects of ACE on oxidative stress, endoplasmic reticulum (ER) stress, cellular death, mRNA expression levels of related genes and protein expressions of related molecular mechanisms in SH-SY5Y human neuroblastoma cells. The half maximal inhibition of enzyme activity (IC_50_) value of ACE was determined as 4.26 mM after 24 h of treatment by MTT assay. We revealed an increase in reactive oxygen species (ROS) production and calcium release. Significant increases were measured in inositol-requiring enzyme 1-alpha (IRE1-α) and binding immunoglobulin protein 90 (GRP90) levels as well as mRNA expression levels of caspase 3, 4 and 9 genes indicating enhanced ER stress. Apoptosis and ER stress-related genes were significantly upregulated at ≥2 mM. Indeed, ACE caused apoptosis and necroptosis while necrosis was not observed. There was a significant increase in the protein level of mitogen-activated protein kinase-8 (MAPK8) at 4 mM of ACE while no change was seen for nuclear factor kappa-B (NF-κB) and tumor necrosis factor-alpha (TNF-α). In conclusion, increased cellular stress markers could be proposed as an underlying mechanism of ACE-induced cell death in neural cells.

## 1. Introduction

Neonicotinoids have taken their place among the most used insecticides worldwide, since they were discovered in the late 1980s [1]. Like nicotine, neonicotinoids act as agonists at nicotinic acetylcholine receptors (nAChRs) on the insect’s central nervous system [2]. Their high affinity for insect receptors allows this group of pesticides to display more selective toxicity to insects than mammals [3]. In 2014, neonicotinoids accounted for more than 25% of the total global insecticide sales [4]. Low toxicity to non-targeted organisms and the environment, high selectivity against insects and a wide variety of application routes are some of the main reasons for the neonicotinoids’ popularity [5]. The hydrophilicity of neonicotinoids enables them to translocate to all tissues of the plant [6]. Even though this offers systemic protection against pests, it might lead to the penetration of the insecticide to non-target organisms and its accumulation in foils.

Acetamiprid (ACE), (E)-N-((6-chloropyridin-3-yl)methyl)-N’-cyano-N-methylacetimidamide, is a commonly used neonicotinoid insecticide [7]. It appears to have a very high activity against *Hemiptera* and *Thysanoptera* like other neonicotinoids; additionally, it shows a great activity against *Lepidoptera* [8]. It is used to control sucking insects on leafy vegetables, citrus and pome fruits, grapes, cottons and many more [9]. It was registered as formulations containing 20% water-soluble powder of ACE and commercialized under different brand names such as MOSPILAN 20, GOLDPLAN 20, HEKPLAN 20, etc., in Turkey, and different concentrations ranging from 0.06 to 0.4 g/L water were recommended for numerous insects due to their life-cycle and agriculture field [10].

Though ACE is considered safe to use [9], there are case reports suggesting that ACE has immunotoxic effects by lowering lymphocyte production and decreasing macrophage functions [7] and has hepatotoxic effects by losing hepatic membrane structure and causing hepatocellular damage [11]. Additionally, ACE causes oxidative stress by decreasing antioxidant enzymes in male mice’s reproductive system [12] and leads to apoptosis in rat adrenal medulla cells [13]. Moreover, it has been reported that ACE exposure could be correlated with neurological symptoms such as short-term memory loss, tremor, headache and fatigue [14]. Since the brain is particularly vulnerable to oxidative stress due to its unique properties such as richness in peroxidable fatty acids, auto-oxidation and modest antioxidant capacity [15], increased cellular stress could cause several neurological symptoms and ultimately diseases.

All in all, the purpose of this study was to investigate the neurotoxic potential of ACE in human bone marrow-derived neuroblastoma (SH-SY5Y) cells, a dopaminergic neuronal cell line, which has expressed human proteins and has been used as an in vitro model for neurotoxicity [16]. In the present study, cytotoxic activity was determined by MTT assay, while the oxidative damage potential was investigated by measuring the ROS production, glutathione (GSH) levels, total antioxidant status (TAS) and total oxidant status (TOS). Intracellular calcium levels were measured using flow cytometry. Apoptotic, necroptotic and necrotic cells were distinguished by flow cytometry using Annexin V/PI and confirmed by fluorescence microscopic imaging. Furthermore, mRNA expression levels of apoptosis- and ER stress-related genes were determined, and protein expressions of related genes were evaluated by either Western blot or enzyme-linked immunosorbent assay (ELISA).

## 2. Materials and Methods

### 2.1. Chemicals

ACE (97%) was kindly gifted from Hektaş Ticaret A.Ş. (Istanbul, Turkey). ELISA kits for GSH, TAS, TOS, IRE1-α, growth arrest- and DNA damage-inducible gene 153 (GADD153), glucose-regulated protein-78 and -90 (GRP78 and GRP90) and caspase-12 (CASP12) from Elabscience Biotechnology Co. Ltd. (Houston, TX, USA), TNF-α from Thermo Fisher Scientific Inc. (KHC3011, CA, USA); FITC Annexin V Apoptosis Detection Kit with PI from BioLegend (San Diego, CA, USA); FluoForte calcium assay kit from Enzo Life Sciences, Inc. (Lausen, Switzerland); 4′,6-diamidino-2-phenylindole (DAPI) from BioFroxx (GmbH, Germany); the primary antibodies, anti-NF-κB (ab6671), anti-TNF-α (ab16502) from Abcam (Cambridge, MA, USA) and anti-MAPK8 (PR5340A) from Thermo Fisher Scientific Inc. (Carlsbad, CA, USA); anti-rabbit IgG HRP-binding antibody from Santa Cruz Biotechnology (Dallas, TX, USA) were purchased. Primers for mRNA expressions were obtained from Sentromer DNA Technologies (Istanbul, Turkey). The total RNA isolation kit and cDNA synthesis kit were from Roche Life Sciences (Penzberg, Germany); SensiFast No-Rox Kit was from Bioline (London, UK); RIPA lysis buffer and luminol reagent were from Santa Cruz Biotechnology (Dallas, TX, USA). Dimethyl sulfoxide (DMSO), sodium dodecyl sulfate (SDS), 3-[4,5-dimethylthiazol-2-yl]-2,5-diphenyl-tetrazolium bromide (MTT) and 2′,7′-dichlorodihydrofluorescein diacetate (H2DCF-DA) were from Sigma Chemical Co. Ltd. (St. Louis, MO, USA). Phosphate-buffered saline (PBS), Dulbecco’s Modified Eagle Medium: Nutrient Mixture F-12 (DMEM F-12) and all other cell culture supplements were from Multicell Wisent (Saint-Jean Baptiste, QC, Canada), and sterile plastic materials were from Corning (Amsterdam, The Netherlands).

### 2.2. Cell Culture Conditions and Treatments

SH-SY5Y cells (CRL-2266) obtained from the American Type Culture Collection (ATCC) were grown in DMEM F-12 supplemented with 10% FBS and penicillin/streptomycin (100 U/100 μg/mL) at 37 °C in a humidified atmosphere with 5% CO_2_. In every 3–4 days when the cells became confluent, the sub-culturing was performed. The cells were seeded at the appropriate densities ranging from 10^4^ to 10^6^ cells for each assay, and incubated overnight for cellular attachment. 

ACE stock solution was prepared by dissolving in DMSO at the final concentration of 100 mM and kept at −20 °C. Serial dilutions from stock solution were immediately made in the range of 0.5–4 mM before use in the cell culture medium, and exposure duration was 24 h for each assay. All treatments were done in triplicates on independent days.

### 2.3. MTT Assay

MTT compound, a pale yellow water-soluble tetrazolium salt, is reduced to pinkish purple insoluble formazan crystals by the succinate dehydrogenase within the mitochondria in viable cells [17]. Briefly, 10^4^ cells/100 µL medium/well were seeded into 96-well plates, and then incubated overnight. The half-descending concentrations starting from 100 mM of ACE were added to wells; additionally, the medium, DMSO (1%) and SDS (1%) were used as growth, negative and positive controls, respectively. The optical densities (ODs) were measured at 590 nm by a microplate spectrophotometer system (Epoch, Germany). Each compound was tested in triplicates in a plate and all ODs were used after subtracting the mean OD of blank wells. The inhibition of cell growth was calculated for each concentration tested using the formula:

Reduction of cell viability (%) = 100−[(_mean_OD of tested concentration/_mean_OD of solvent control)] × 100 (1)

Then, the half maximal inhibitory concentration (IC_50_) was calculated according to the formula of logarithmic curve of inhibition-concentration graphic.

### 2.4. Oxidative Stress Parameters

The cells naturally have a balance, also known as redox homeostasis, between oxidants (i.e., ROS) and antioxidant defense systems (i.e., GSH). When ROS generation overwhelms the natural antioxidant defenses of cells and the imbalance occurs, the ultimate result is oxidative stress. 

#### 2.4.1. Flow Cytometry

The cellular ROS generation was measured by H_2_DCF-DA, which is attributed as a direct indicator of redox homeostasis [18]. Briefly, 10^5^ cells/2 mL medium/well were seeded into 6-well plates, and then incubated overnight. ACE (0.5–4 mM) and DMSO (1%), negative control, were added to wells. At the end of the 24 h treatment, the cells were detached and re-suspended in 200 µL PBS containing 20 μM H_2_DCF-DA and incubated at room temperature for 30 min in the dark. Fluorescence intensities of 10,000 gated events per sample were measured at 488 nm on an ACEA NovoCyte flow cytometer (San Diego, CA, USA). The results were expressed as the median fluorescence intensity (MFI), which demonstrates the shift in fluorescence intensity of a cell population in a histogram. The alterations in cellular ROS production were demonstrated as the percentage of MFI (MFI%), which is calculated by dividing the MFI of the concentration tested to the control and multiplied by 100. 

#### 2.4.2. GSH Content Determination

The GSH levels were measured by an ELISA kit that has pre-coated plates with human GSH antibody. Briefly, 10^6^ cells/5 mL medium were seeded into 25 cm^2^ culture flasks, and then incubated overnight. ACE (0.5–4 mM) and DMSO (1%), negative control, were added to flasks. At the end of the 24 h treatment, the cells were detached with trypsin-EDTA, washed in ice-cold PBS, and diluted to ~10^6^ cells/mL in PBS. Finally, the cells were repeatedly frozen and thawed three times to lysis, and an ELISA assay was performed according to the manufacturer’s instructions. The Bradford method [19] was used to measure the amount of protein in 106 cells, and the results were expressed as μg/mg protein using a standard calibration curve.

#### 2.4.3. TAS and TOS Analysis

TAS and TOS were measured by an ELISA kit according to the manufacturer’s instructions. Briefly, 10^6^ cells/5 mL medium were seeded into 25 cm^2^ culture flasks, and then incubated overnight. ACE (0.5–4 mM) and DMSO (1%), negative control, were added to flasks. At the end of the 24 h treatment, an ELISA assay was performed, and the results were expressed as U/mL for both TAS and TOS assays. The oxidative stress index (OSI), calculated as the ratio of TOS to TAS, was used as a more precise indicator of oxidative stress [20]. 

### 2.5. Calcium Mobilization

Fluorescent calcium probes are widely used for the detection of calcium release in the nucleus and cytosol as well as the changes in calcium concentrations in organelles [21]. The intracellular calcium was measured using the FluoForte Calcium Assay Kit according to the manufacturer’s instructions with slight modifications. Briefly, 10^5^ cells/2 mL medium/well were seeded into 6-well plates, and then incubated overnight. ACE (0.5–4 mM) and DMSO (1%), negative control, were added to wells. The reagents were prepared as directed and equilibrated to room temperature prior to use. At the end of the 24 h treatment, the cells were detached with trypsin-EDTA, washed in ice-cold PBS twice, and re-suspended in FluoForte dye-loading solution (250 μL). The cells were subsequently incubated at 37 °C for 45 min and then at room temperature for 15 min in the dark. Finally, fluorescence intensities of 10,000 gated events per sample were measured at 488 nm on ACEA NovoCyte flow cytometer (San Diego, CA, USA). The alterations in intracellular calcium were demonstrated as the percentage of MFI (MFI%), which is calculated by dividing the MFI of the concentration tested to the control and multiplied by 100. 

### 2.6. ER Stress

ER stress is an early indicator of disrupted cellular processes, since the accumulation of the misfolded proteins in the ER lumen may lead to impairment of cellular functions [22]. IRE1-α, GADD153, GRP78, GRP90 and CASP12 were measured by an ELISA kit according to the manufacturer’s instructions. Briefly, 10^6^ cells/5 mL medium were seeded into 25 cm^2^ culture flasks, and then incubated overnight. ACE (0.5–4 mM) and DMSO (1%), negative control, were added to flasks. At the end of the 24 h treatment, an ELISA assay was performed, and the results were expressed as ng/mL.

### 2.7. Apoptosis/Necrosis

Apoptotic and necrotic potential of ACE was determined by using Annexin V/PI by flow cytometer, and the results were also confirmed by fluorescent microscopic imaging. 

#### 2.7.1. Flow Cytometry

Apoptotic and necrotic cells are easily distinguished by Annexin V co-staining with PI by a flow cytometer [23]. Briefly, 10^5^ cells/2 mL medium/well were seeded into 6-well plates, and then incubated overnight. ACE (0.5–4 mM) and DMSO (1%) as a negative control were added to wells. At the end of the 24 h treatment, the cells were detached with trypsin-EDTA, washed into cell staining buffer twice and re-suspended in 100 μL binding buffer. Annexin V (1 μL) and PI (1 μL) were added, mixed well and incubated at room temperature for 15 min in the dark. Finally, binding buffer (200 μL) was added and fluorescence intensities of 10.000 gated events per sample were measured at 488 nm on an ACEA NovoCyte flow cytometer (San Diego, CA, USA). The results were expressed as the percentage of the total number of cells obtained from quadrants.

#### 2.7.2. Fluorescent Microscopic Imaging

Apoptotic and necrotic cells were visualized by fluorescent microscopic imaging after Annexin V/PI staining and counterstaining with DAPI. The cells were seeded at low density to avoid higher backgrounds. Briefly, 10^4^ cells/500 µL medium/well were seeded into 24-well plates, and then incubated overnight. ACE (0.5–4 mM) and DMSO (1%) as a negative control were added to wells. At the end of the 24 h treatment, the cells were washed into cell staining buffer twice. Annexin V, PI and DAPI (1 μL of each) were added into binding buffer (300 μL), and then incubated at room temperature for 15 min in the dark. Finally, the cells were observed at 488 nm and 405 nm with an Epi-illumination fluorescence microscope (Nikon, Eclipse-Ti-U) and captured with a Nikon camera (DSRi1). A hundred cells were scored based on DAPI staining and discriminated as apoptotic (Annexin V^positive(pos)^/PI^negative(neg)^), necroptotic (Annexin V^pos^/PI^pos^) and necrotic (Annexin V^neg^/PI^pos^). The results were expressed as the percentage of the total number of cells.

### 2.8. mRNA Expression Levels of Apoptosis- and ER Stress-Related Genes

Gene expression analysis of *Bax, Bcl-2, p53, MAPK8, NF-κB, TNF-α, CASP3, CASP4* and *CASP9* were determined on the Roche Real Time LightCycler^®®^480 platform (Mannheim, Germany) as previously described by Karaman and Ozden [24] with minor modifications. Briefly, 5 × 10^5^ cells/5 mL medium were seeded into 25 cm^2^ flasks, and then incubated overnight. ACE (0.5–4 mM) or DMSO (1%), negative control, were added into the flasks and the cells were incubated for 24 h before total RNA were extracted. cDNA was synthesized from isolated RNA, and real-time PCR reactions were performed using SensiFast No-Rox Kit with 50 ng cDNA in the final mix. The primer sequences and the annealing temperatures of the genes are provided in Table 1. The cycle threshold (Ct) of real-time PCR specific for the apoptosis-related genes and *β-actin*, the housekeeping gene, were determined; and the relative expressions were evaluated by the comparative Ct method.

### 2.9. Western Blot Analysis of NF-κB and MAPK8

The alterations of protein expression levels of NF-κB and MAPK8 were evaluated by Western blot analysis using the BioRad Mini Protean Tetra Cell wet transfer Western blotting system (Bio-Rad). Once treated with ACE (0.5–4 mM) or DMSO (1%) for 24 h, the cells were lysed using RIPA lysis buffer. Equivalent amounts of 30 μg protein were separated on polyacrylamide gel (10%) and transferred onto the polyvinylidene fluoride (PVDF) membranes. The membranes were blocked for 2 h at room temperature in skim milk (5%) in tris-buffered saline with Tween 20 (TBST) and incubated overnight with primary antibody diluted 1:1000 at 4 °C. Then, the blots were washed with TBST and incubated with a secondary antibody (anti-rabbit IgG, dilution of 1:5000) conjugated with horseradish peroxidase for 1 h at room temperature. JNK-bounded membranes were stripped using stripping buffer (Fisher Scientific Inc., Carlsbad, CA, USA) and then re-probed with β-actin primary antibody. After that, the bands were visualized using a luminol reagent by Kodak Molecular Imaging Software (Kodak Company, New York, NY, USA). ImageJ Software (National Institutes of Health, Baltimore, MD, USA) was used for densitometric analysis of the immunopositive band, and the results calculated as arbitrary units were normalized to the density of β-actin bands.

### 2.10. Protein Expression Levels of TNF-α

The TNF-α protein levels were measured by an ELISA kit according to the manufacturer’s instructions. Briefly, 10^6^ cells/5 mL medium were seeded into 25 cm^2^ culture flasks, and then incubated overnight. ACE (0.5–4 mM) and DMSO (1%) as a negative control were added to flasks. At the end of the 24 h treatment ELISA assay was performed, and the results were expressed as pg/mL.

### 2.11. Statistical Analysis

Data were expressed as mean ± standard deviation (SD). Statistical analyses were carried out with one-way ANOVA and Post Hoc Dunnet t-test using SPSS v.20 (IBM SPSS Inc., New York, NY, USA). A two-tailed *p* < 0.05 was considered to indicate a statistically significant difference.

## 3. Results

### 3.1. Effects of ACE on Cell Viability in SH-SY5Y Cells

**Figure 1:**
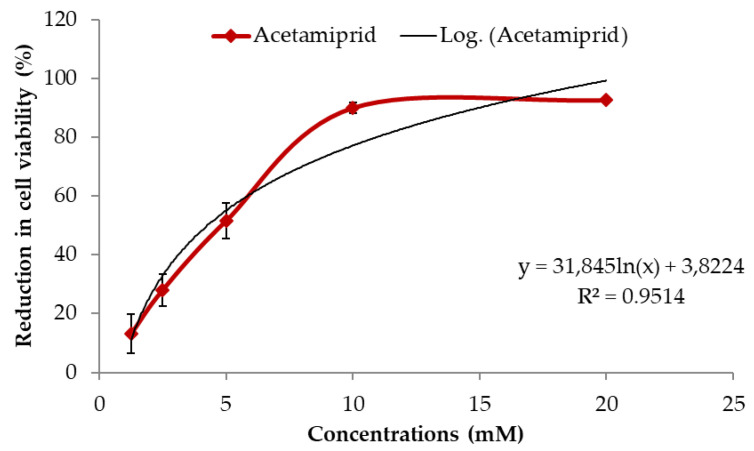
Effects of ACE (0–20 mM) on cell viability by MTT assay (A) in SH-SY5Y cells after 24 h of treatment. IC50 value of ACE was calculated as 4.26 mM according to the formula of logarithmic curve (black line). Data are presented as mean ± SD.

IC50 value of ACE was calculated as 4.26 mM by MTT assay in SH-SY5Y cells. As shown in Figure 1, a dose-dependent reduction in cell viability was observed up to 10 mM concentrations, and ≥90% cells died in the higher concentrations (10 and 20 mM).

### 3.2. Effects of ACE on Oxidative Stress in SH-SY5Y Cells 

**Figure 2:**
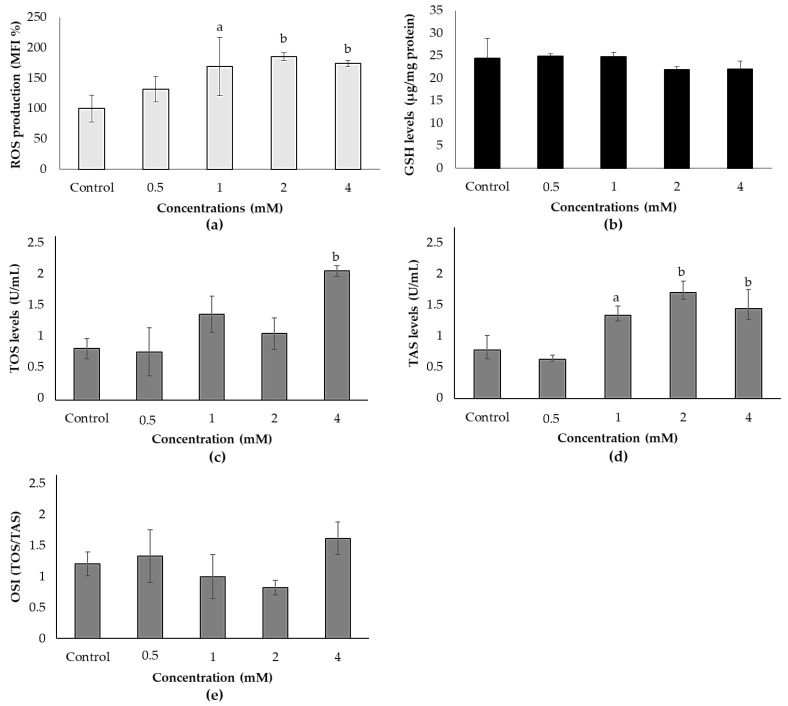
Effects of ACE on (**a**) ROS production; (**b**) GSH levels; (**c**) TOS levels, (**d**) TAS levels and (**e**) OSI in SH-SY5Y cells after 24 h of treatment. Data are presented as mean ± SD; and statistically significant changes are indicated as a: *p* < 0.05 and b: *p* < 0.01.

H_2_-DCFDA results indicated that ACE increased the ROS production in a dose-dependent manner. ROS production was significantly induced in 1, 2 and 4 mM concentrations of ACE at the rates of 69.05%, 85.31% and 74.05%, respectively, compared to the control (Figure 2a). Similarly, TOS levels increased in a dose-dependent manner; however, statistical significance was attained only at the highest concentration (*p* < 0.01) (Figure 2c). TAS levels also significantly increased at ≥1.2-fold in 1, 2 and 4 mM concentrations of ACE (Figure 2d). Similar to GSH levels (Figure 2b), OSI did not change at any concentrations tested (Figure 2e) despite the increases in TOS and TAS levels that were measured.

### 3.3. Effects of ACE on Calcium Mobilization in SH-SY5Y Cells

**Figure 3:**
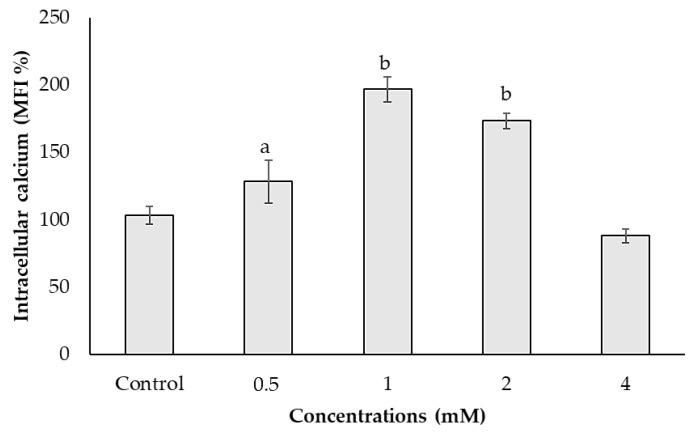
Effects of ACE on intracellular calcium in SH-SY5Y cells after 24 h of treatment. Data are presented as mean ± SD; and, statistically significant changes are indicated as a: *p* < 0.05 and b: *p* < 0.01.

Intracellular calcium significantly increased at 0.5, 1 and 2 mM concentrations of ACE at the rates of 28.52%, 97.03% and 73.52%, respectively, compared to the control. Furthermore, calcium content decreased about 20% at 4 mM concentration compared to the control (Figure 3).

### 3.4. Effects of ACE on Endoplasmic Reticulum Stress in SH-SY5Y Cells

**Figure 4:**
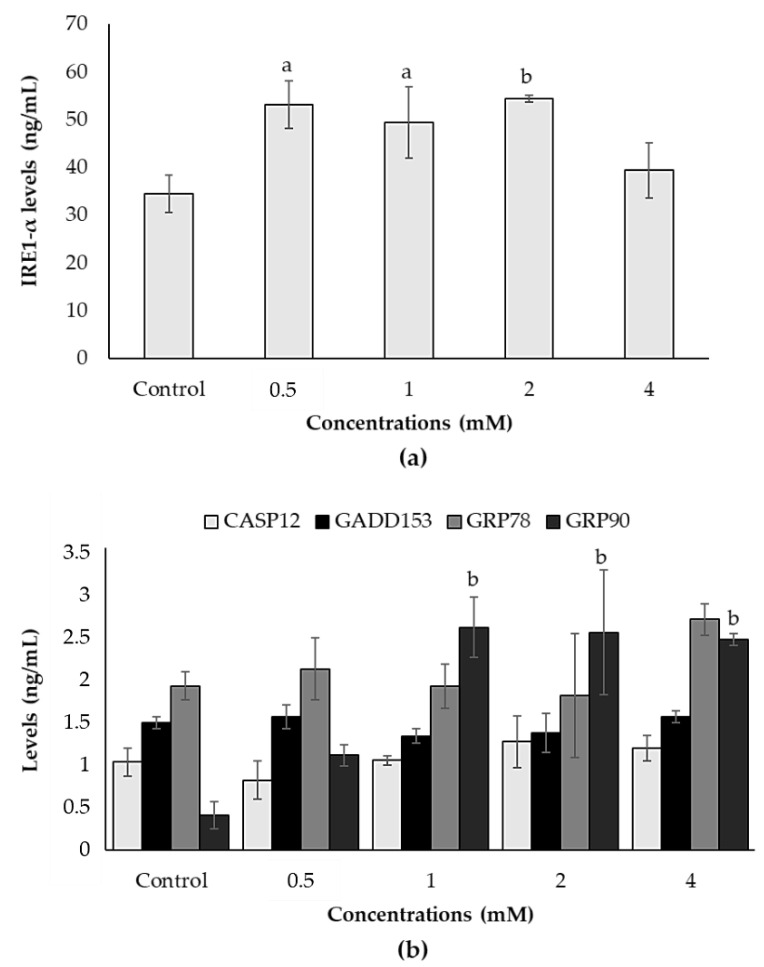
Effects of ACE on endoplasmic reticulum stress in SH-SY5Y cells after 24 h of treatment. (**a**) IRE1-α and (**b**) GRP90 levels were significantly increased with ACE treatment. Data are presented as mean ± SD; and statistically significant changes are indicated as a: *p* < 0.05 and b: *p* < 0.01.

IRE1-α levels significantly increased at 0.5, 1 and 2 mM concentrations of ACE at the levels of 53.24 ng/mL, 49.43 ng/mL and 54.35 ng/mL, respectively, compared to the control (34.51 ng/mL) (Figure 4a). Additionally, GRP90 levels significantly increased at 1, 2 and 4 mM concentrations of ACE at the levels of 2.58 ng/mL, 2.52 ng/mL and 2.43 ng/mL, respectively, compared to the control (0.41 ng/mL). However, CASP12, GADD153 and GRP78 levels did not change in any concentrations tested (Figure 4b).

### 3.5. Effects of ACE on Apoptosis and Necrosis in SH-SY5Y Cells

**Figure 5:**
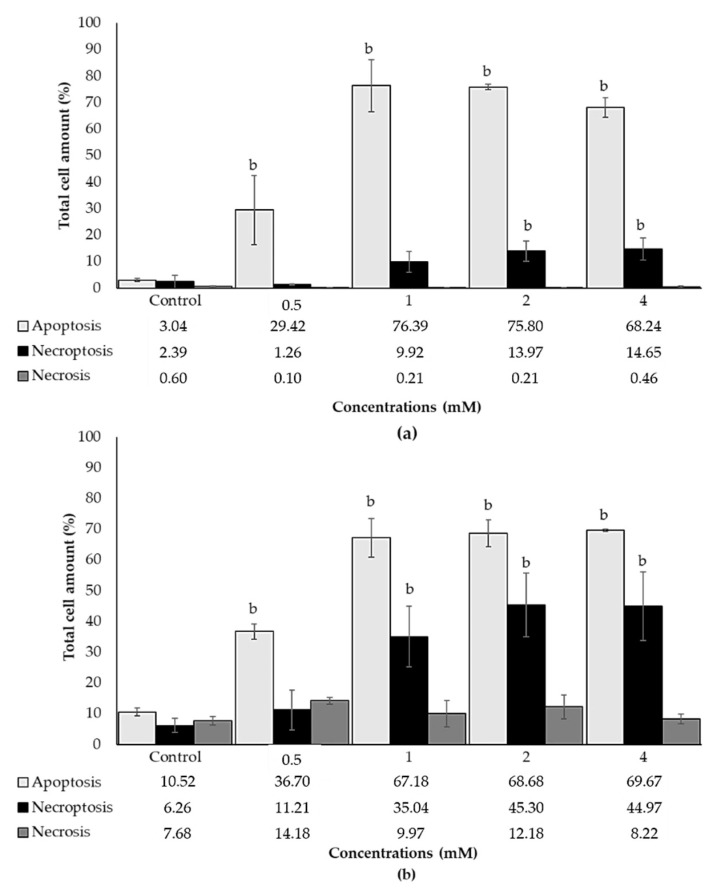
Apoptotic and necrotic effects of ACE measured by (**a**) flow cytometer and (**b**) fluorescent microscopic imaging in SH-SY5Y cells after 24 h of treatment. Apoptotic cells were expressed as the cell population of Annexin V^pos^/PI^neg^, necroptotic cells were expressed as the cell population of Annexin V^pos^/PI^pos^ and necrotic cells were expressed as the cell population of Annexin V^neg^/PI^pos^. Data are presented as mean ± SD; and statistically significant changes are indicated as b: *p* < 0.01.

The cellular death pathway was determined by Annexin V/PI staining by a flow cytometer and fluorescent microscopic imaging. ACE significantly caused apoptotic cell death between the range of 29.42% and 76.39% in all concentrations tested. Although necroptosis was induced in a dose-dependent manner, significant changes were observed at 2 and 4 mM concentrations of ACE at the rates of 13.97% and 14.65%, respectively, whereas necrosis was not induced in any concentrations tested (Figure 5a). Additionally, semi-quantitative analysis obtained from fluorescent microscopic images showed that ACE induced apoptosis between the range of 36.70% and 69.67% in all concentrations tested. Additionally, ACE induced necroptosis between the range of 35.04% and 45.30% at higher concentrations while necrosis was not induced (Figure 5b). Even though the necroptotic and necrotic cell rates were higher in fluorescence microscopic analysis, apoptotic cell rate was similar in both flow cytometric and fluorescence microscopic analyses. The representative fluorescent microscopic images are provided in Supplementary Material (Figure S1).

### 3.6. Effects of ACE on mRNA Expression Levels of Apoptosis and ER Stress Related Genes in SH-SY5Y Cells

**Figure 6:**
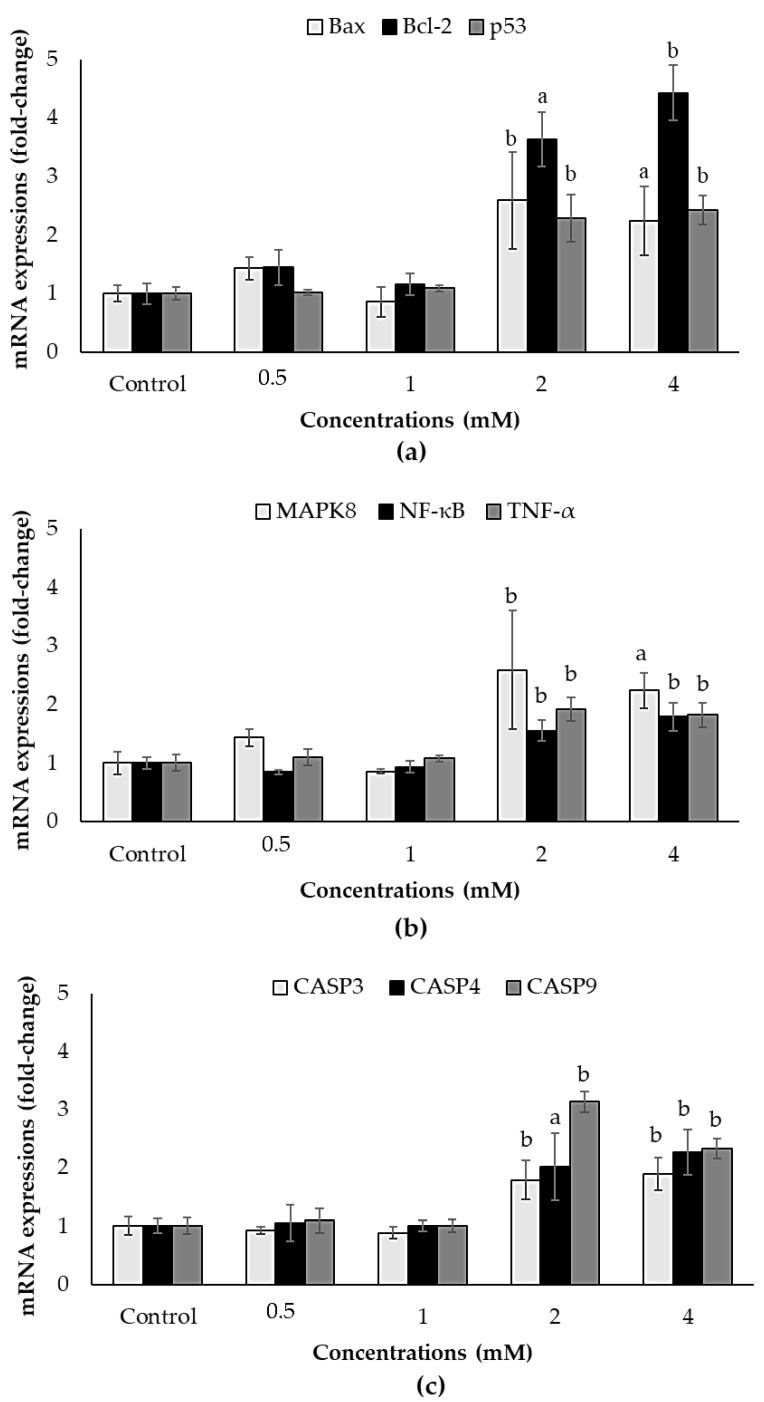
Effects of ACE on relative mRNA expression levels of (**a**) *Bax, Bcl-2* and *p53*, (**b**) *MAPK8, NF-κB* and *TNF-α*, (**c**) *CASP3, CASP4* and *CASP9* in SH-SY5Y cells after 24 h exposure. Data are presented as mean ± SD; and statistically significant changes are indicated as a: *p* < 0.05 and b: *p* < 0.01.

The most apparent alteration occurred in *Bcl-2* with 3.63-fold and 4.42-fold upregulation at 2 and 4 mM concentrations of ACE, respectively, compared to the control (Figure 6a). Additionally, the two highest concentrations (2 and 4 mM) of ACE significantly increased the mRNA expression levels of *Bax* (≥2.24-fold), *p53* (≥2.29-fold), *MAPK8* (2.36-fold), *NF-κB* (≥1.56-fold), *TNF-α* (≥1.82-fold), *CASP3* (≥1.80-fold), *CASP4* (≥2.02-fold) and *CASP9* (≥2.34-fold) (Figure 6a–c).

### 3.7. Effects of ACE on Protein Expression Levels in SH-SY5Y Cells

**Figure 7:**
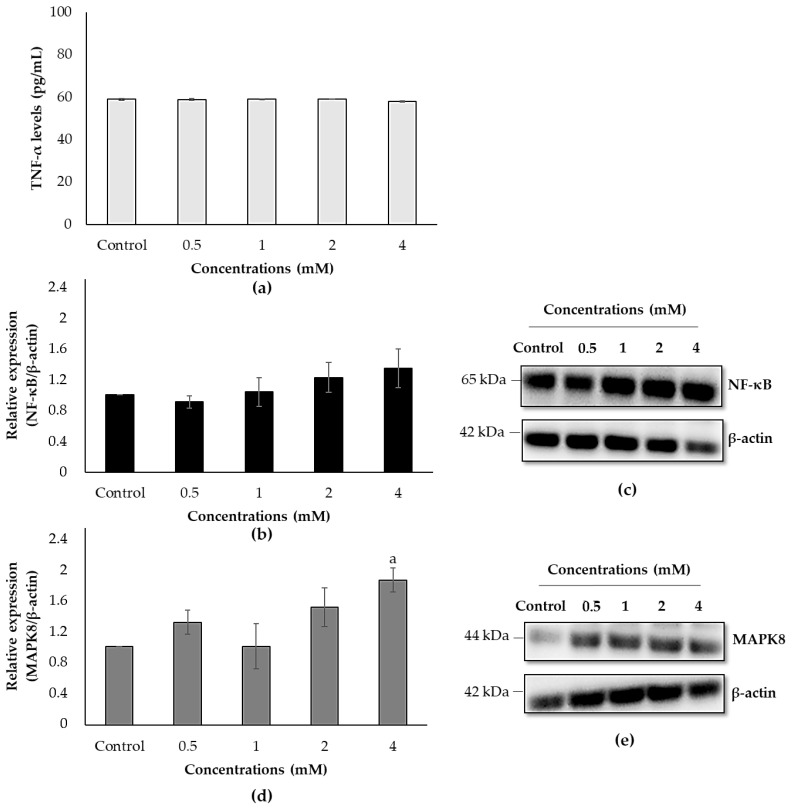
Effects of ACE on (**a**) TNF-α levels, (**b**) NF-κB and (**d**) MAPK8 protein expressions in SH-SY5Y cells after 24 h exposure. Representative membrane images of (**c**) NF-κB and (**e**) MAPK8. Data are presented as mean ± SD; and statistically significant changes are indicated as a: *p* < 0.05.

TNF-α levels were measured by an ELISA assay, and NF-κB and MAPK8 protein expressions were detected by Western blot analysis to identify the ultimate effect of ACE on the apoptosis pathway. TNF-α levels did not change in any concentrations tested (Figure 7a), and NF-κB protein expression slightly increased in 4 mM of ACE (Figure 7b,c). Although there was a dose-dependent-like increment in MAPK8 protein expressions, 2 and 4 mM of ACE increased the MAPK8 level by 1.37-fold (*p* = 0.084) and 1.48-fold (*p* = 0.023), respectively (Figure 7d,e). All Western blot images and densitometric analyses are presented in Supplementary Material (Figures S2–S4).

## 4. Discussion

Neonicotinoids, which act as nicotinic acetylcholine receptor agonists, are widely used insecticides introduced to the global market with growing importance [32]. Neonicotinoids are thought to have a selective binding affinity in the central nervous system of insects and low toxicity on vertebrate species due to poor penetration of the mammalian blood–brain barrier, which is the reason for their popularity as nAChR agonists [3,33]. However, the high water solubility of neonicotinoids also leads the compounds to be extremely mobile, and so their residues being easily transferred to aquatic ecosystems resulted in accumulation in soil or water [34,35]. ACE is one of the most widely used neonicotinoids globally utilized to protect crops from insects [33]. It belongs to a new class of insecticides that is distributed throughout the body, such as in the liver, kidney, adrenal and thyroid glands, by reaching a high concentration [36].

ACE can potentially accumulate in the brain of murine, rats and also the human body [37]. It has been well investigated that ACE has hepatotoxic, reproductive and developmental toxic effects in in vivo animal studies [11,38,39]. It has been reported that ACE could cause several alterations in brain functions in rats [40] and neurological symptoms in humans [14]. Since inhalation is one of the main exposure routes for neonicotinoids such as ACE, it could be absorbed in the lung and then ACE and its metabolites could reach the brain passing the blood–brain barrier [41]. Therefore, there is growing evidence about neurotoxic effects of ACE and there have been a few studies of neonicotinoid-induced neurotoxicity in human. However, mechanisms of action have not been fully elucidated, so the objective of the present study was to determine ROS generation, apoptosis and ER stress parameters for potential human neurotoxicity of ACE in SH-SY5Y cells.

In the study, ACE decreased cell viability in a dose-dependent manner and the IC_50_ value was calculated as 4.26 mM. The results have been supported by other in vitro studies in which the IC_50_ value of ACE was determined as 2.16 and 6.68 mM in SH-SY5Y [42] and SK-N-SH human neuroblastoma cells [43], respectively. On the other hand, the IC_50_ values of ACE have also been observed less than 1 mM in different cell lines such as human lung fibroblast IMR-90 [44], human placenta trophoblast HTR-8/SVneo [45] and rat adrenal gland pheochromocytoma PC-12 [13] cells. In view of these data, it can be suggested that human neural cells are more resistant to the cytotoxic effects of ACE.

It has been shown that ACE could lead to oxidative damage by producing ROS in various bacteria [46,47,48] and resulting in disturbance of oxidative status, and loss of mitochondrial membranes integrity in the rat reproductive tract and brain. It has also been reported that the insecticide ACE increased ROS levels, induced lipid peroxidation and decreased levels of antioxidant defense system components including GST, CAT and SOD activity [13,45,49]. In the present study, ACE induced oxidative stress in SH-SY5Y cells by increasing ROS, TOS and TAS levels, but in contrast, GSH levels and OSI did not change after ACE exposure for 24 h. Parallel to our results, Gomez et al. (2020) [45] observed that GSH content showed no changes while ROS production induced drastically in the studied concentrations (0.1–100 μM).

The alterations in redox status could also cause the onset of apoptosis, and it has been reported that the induction of apoptosis in ACE-mediated toxicity was shown in many studies [13,45,46]. Among the studied genes, *Bcl-2* has an anti-apoptotic function; however, *Bcl-2* overexpression was also observed after exposure of ACE in SH-SY5Y cells. Besides its many vital functions called the “guardian of the genome”, *p53* could induce apoptotic cell death by upregulation of *Bax*, as well as inhibition of *Bcl-2* expression via both the extrinsic and the mitochondrial (intrinsic) pathways [50]. The Bax/Bcl-2 ratio, which is the balance between pro-apoptotic *Bax* and anti-apoptotic *Bcl-2,* is known as a key factor in regulating the apoptosis, and an increased Bax/Bcl-2 ratio indicates the promotion of cell death [51]. Because of the upregulation of *Bcl-2*, an increase has not been observed in this ratio. In addition, members of the Bcl-2 family proteins such as Bcl-2 and Bax also modulate the mitochondrial membrane potential (MMP) in apoptosis through the upregulation of Bax and disruption of the MMP via imbalance of the Bcl-2/Bax ratio leading to activation of CASP3 and CASP9, which are responsible for executing cell death [52]. Furthermore, p53 and ROS play a vital role in mitochondrial damage, causing a rise in intracellular Ca^2+^ concentration and finally apoptosis initiation [53]. 

In the present study, ACE induced apoptosis and upregulated apoptosis-related genes such as *Bax, p53, CASP3 and CASP9*. In harmony with these genes, cell death profiles evaluated by Annexin V and PI staining clearly indicate that ACE induced cell death via apoptosis rather than necrosis. Even though the necroptotic and necrotic cell rates were higher in fluorescence microscopic analysis, apoptotic cell rate was similar in both flow cytometric and fluorescence microscopic analyses. One possible explanation of this is that higher background and/or cellular debris could affect the semi-quantitative scoring. On the other hand, flow cytometry is a more sensitive and accurate instrument as it can sort cells based on size and granularity and less affected by cellular bulk and debris. It has also been observed that intracellular calcium level slightly decreased when 4 mM of ACE was administrated. Considering the cell death in this concentration, it is conceivable that cells could undergo the blebbing phase with the reduced calcium content. 

Stress factors such as the alterations in redox state or Ca^2+^ concentrations decrease the protein folding capacity of the ER, causing the accumulation and aggregation of unfolded proteins and triggering the unfolded protein response (UPR) called ER stress. Several studies have revealed that ER stress is related to environmental chemical-induced toxicity and oxidative stress, and that mitochondrial dysfunction and apoptosis contribute to ER stress with close interactions [54,55,56]. In connection with apoptosis induction and disruption of calcium storage, it was also observed that ACE treatments increased the amount of IRE1-α, GRP90 and CASP4, which are the main modulators of ER stress parameters. Unfolded protein accumulations in the ER activate ER-stress sensors, and *IRE1-α* overexpression induces the UPR [57]. As the key indicators of ER stress, GRP90, one of the cytoprotective ER chaperones, increases the protein folding capacity. The UPR is primarily responsible for pro-survival function by increasing the expression levels of ER chaperones. It could be suggested as a result of our study that the upregulation of GRP90 was due to ER stress response after ACE exposure. It is also known that CASP4 activation is also one of the hallmarks in the ER stress-induced apoptosis [58]. 

With the generation of ROS and the release of calcium from the ER, inflammation is also related to the activation of NF-κB and MAPK [59]. Previous studies have reported that the MAPK signaling pathway regulates various cellular processes such as apoptosis, mitochondrial dysfunction and ER stress in the chemical-induced toxicity [55,60]. Therefore, to further explore, protein expression levels of MAPK8 were evaluated using Western blot and it was observed that MAPK8 increased after 2 and 4 mM of ACE treatments. Furthermore, it has been known that NF-κB is vital for converting ER stress signals to induce the inflammatory processes, and dysregulation of calcium levels contributes to the activation of NF-κB in response to ER stress [61]. The present study showed that ACE slightly induced NF-κB in protein levels.

The crosstalk between ER and mitochondria is regulated by calcium-dependent processes. Elevated levels of ROS generation in the mitochondria cause the release of calcium from ER by triggering calcium-releasing channel in the ER membrane, and high Ca^2+^ concentrations also transduce an apoptotic signal and trigger cell death [62,63]. Hence, ER and oxidative stress collaborate to induce apoptosis either by affecting normal Ca^2+^ flux from ER to mitochondria or altering Ca^2+^ signaling due to the increased ROS levels resulting in abnormal mitochondrial homeostasis [64]. In the present study, ACE was shown to cause an increase in apoptosis rate through the augmentation of intracellular Ca^2+^ levels, induction of ER stress and ROS generation. Moreover, connections between the UPR and apoptotic machinery by activation of MAPK8, which is known to regulate Bcl-2 proteins, have been reported [65]. In addition, it has been reported that Bax interacts directly with IRE1, and p53 also regulates ER function during ER stress [66]. Stress signals are transferred from the ER to mitochondria, and ER stress-induced apoptosis, like mitochondrial pathway-mediated apoptosis, is also arranged by the Bcl-2 family [67]. Therefore, it could be suggested that apoptosis through ER stress constitutes mechanisms underlying ACE-induced neurotoxicity via upregulated *Bax* and *p53* by activating MAPK8.

Once and for all, pesticides in the market should be re-evaluated periodically to ensure that they continue to meet the appropriate safety standards. The diversity of health problems caused by pesticides is broad, including neurotoxicity, immunotoxiciy, carcinogenicity, mutagenicity and endocrine disruption. Considering the mechanism of action of ACE, it is quite possible to observe neurotoxic effects compatible with clinical case reports mentioned before. It is worth mentioning that ACE is relatively non-persistent and its rapid degradation will reduce its accumulation in soil and penetration into groundwater [9]. Even though ACE is considered to be a selective and relatively safe choice, there is always a risk to human health mainly via residues in food products. The European Union Commission (EU 2019/88) [68] amendment to Regulation (EC) No.396/2005 reassessed and renewed the maximum residue levels (MRLs) of ACE in numerous food products including fruits, vegetables, nuts, beans, herbs, spices and meat and dairy products varying from 0.01 mg/kg to 3 mg/kg. It should be noted that the highest residue levels were detected in olives, salad plants, small berries and edible herbs such as rosemary, sage, thyme, etc. Since the Mediterranean diet is rich in vegetables, fruits, herbs, spices and extra virgin olive oil, and is therefore highly recommended in order to avoid cardiovascular diseases, neurodegenerative diseases, diabetes and obesity [69], individuals following the Mediterranean diet are more susceptible to ACE exposure. The EPA established no observed adverse effect level (NOAEL) of 10 mg/kg and a lowest observed adverse effect level (LOAEL) of 30 mg/kg based on acute mammalian neurotoxicity study in rats due to evidence of decreased locomotor activity [9]. In 2011, The Food and Agriculture Organization/World Health Organization (FAO/WHO) Joint Committee established an acceptable daily intake (ADI) of 0–0.07 mg/kg bw on the basis of the NOAEL of 7.1 mg/kg bw per day obtained from a 2-year study of toxicity and carcinogenicity in rats regarding the clinical signs and hepatotoxicity [70]. Moreover, the committee stated acute reference dose (ARfD) of 0.1 mg/kg bw regarding a NOAEL of 10 mg/kg bw obtained from an acute neurotoxicity study in rats. Later, in 2014, EFSA recommended a four-times lower acute reference dose (ARfD) of 0.025 mg/kg bw based on its reasoned opinion on developmental neurotoxicity of ACE [71]. In the present study, we observed that ACE exerts neurotoxicity at the concentration range of 0.5–4 mM in human neuroblastoma cells after 24 h exposure. The lowest concentration in which we acquired the toxicity signals was 0.5 mM, which is approximately equal to 0.11 mg/mL (111.35 mg/L = 111.35 ppm = 111.35 mg/kg) and ten-times higher than NOAEL. Considering the recent MRL, ADI and ARfD values, neurotoxicity is not an issue via dietary exposure in the general population; however, neurotoxicity can be observed in accidental or deliberate acute overdose. Furthermore, in the literature, there were no detailed studies on neural cells focused on molecular mechanisms and/or neuron histopathology.

## 5. Conclusions Remarks

Based on our experimental studies, collective data indicate that ACE-mediated oxidative stress led to SH-SY5Y cells apoptosis with ER stress-triggered signaling pathways. Our findings clearly demonstrate the implications of oxidative stress, apoptosis and ER stress in the neurotoxicity of ACE. Besides, the nervous system is quite complex and comprises different cell types, so further studies and new in vitro systems that reflect the complexity of the nervous system are needed to elucidate the neurotoxic effects of ACE on humans.

## Figures and Tables

**Table 1:** Primers used real-time PCR analysis of selected genes and the corresponding annealing temperatures (Ta, °C).

Gene	Primer Sequences (5′–3′)	Ta	Reference
*Bax*	F: ACCAAgAAgCTgAgCgAgTATC R: ACAAAgATggTCACggTCTgCC	60	[25]
*Bcl-2*	F: TgTggCCCAgATAggCACCCAg R: ACTTCgCCgAgATgTCCAgCCAg	65	[25]
*p53*	F: AgAgTCTATAggCCCACCCC R: GCTCgACgCTAggATCTgAC	61	[25]
*MAPK8*	F: TTggAACACCATgTCCTgAA R: ATgTACgggTgTTggAgAgC	57	[26]
*NF-κB*	F: CACTgCTCAggTCCACTgTC R: CTgTCACTATCCCggAgTTCA	61	[27]
*TNF-α*	F: TTCTgTCTACTgAACTTgggggTgATCggTCC R: gTATgAgATAgCAAATCggCTgACggTgTggg	60	[28]
*CASP3*	F: gCTATTgTAggCggTTgT R: TgTTTCCCTgAggTTTgC	53	[25]
*CASP4*	F: AAgAgAAgCAACgTATggCAggAC R: ggACAAAgCTTgAgggCATCTgTA	62	[29]
*CASP9*	F: ACCAgAgATTCgCAAACCAg R: TCACCAAATCCTCCAgAACC	57	[30]
*β-actin*	F: AACTACCTTCAACTCCAT R: TgATCTTgATCTTCATTgTg	48	[31]

## Data Availability

All the data presented in this study are included in the article.

## Supplementary Materials

The following are available online at https://www.mdpi.com/article/10.3390/biology10090820/s1, Figure S1. Representative fluorescence microscopic images after Annexin V/PI staining and counterstaining with DAPI of SH-SY5Y cells exposed to ACE for 24 h, Figure S2. Representative images of whole nitrocellulose membrane in Western blot, Figure S3. Images of NF-κB protein bands, densitometric analysis of relative NF-κB protein expression levels and descriptives of densitometric analysis of relative NF-κB protein expression, Figure S4. Images of MAPK8 protein bands, densitometric analysis of relative MAPK8 protein expression levels and descriptives of densitometric analysis of relative MAPK8 protein expression.
