# Supplementary material for: Cellular Stress Pathways Are Linked to Acetamiprid-Induced Apoptosis in SH-SY5Y Neural Cells
Source: Biology (Basel). 2021 Aug 24;10(9):820. doi: 10.3390/biology10090820 (PMC8467785; doi:10.3390/biology10090820)
Supplement: Supplementary file 1 [file biology-10-00820-s001.zip › biology-1209475-SI.pdf]

Supplementary Materials

Cellular Stress Pathways are Linked to Acetamiprid Induced Apoptosis in SH-SY5Y Neural Cells

Ezgi Öztaş, Mehtap Kara, Tuğçe Boran, Enes Bişirir, Ecem Fatma Karaman, Engin Kaptan and Gül Özhan

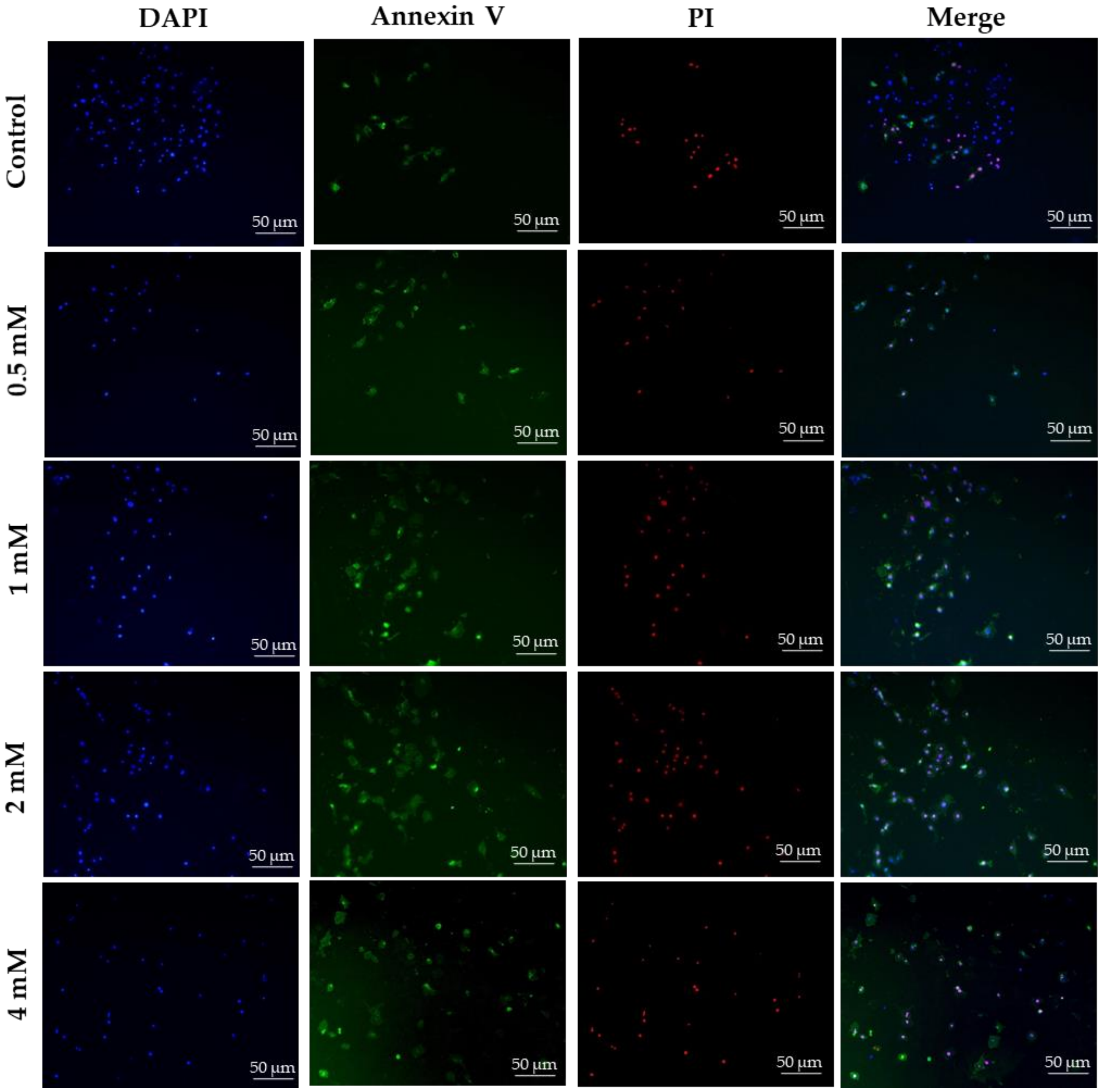

Figure S1. Representative fluorescence microscopic images after Annexin V/PI staining and counterstaining with DAPI of SH-SY5Y cells exposed to ACE for 24 h. DAPI (blue) indicates the nucleus of the cells. Cells stained for Annexin V are green, cells stained for PI are red, and cells stained for both are merged and shown as purple. Cells were discriminated as apoptotic (Annexin V^{pos}/PI^{neg}), necroptotic (Annexin V^{pos}/PI^{pos}) and necrotic (Annexin V^{neg}/PI^{pos}). Images were captured using an ×10 objective.

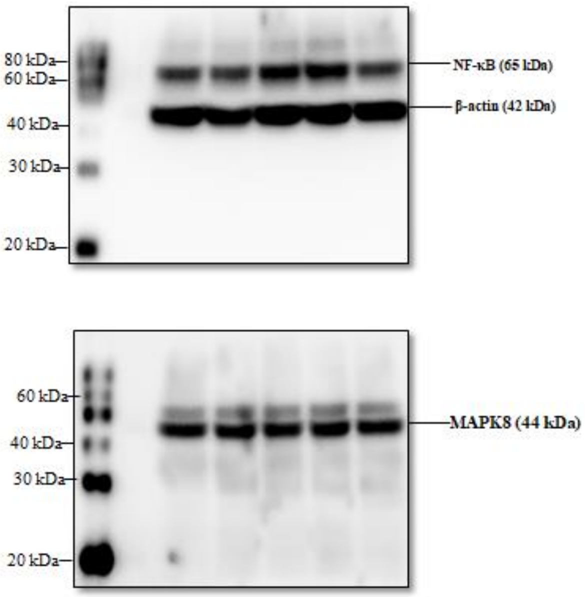

Figure S2. Representative images of whole nitrocellulose membrane showing protein weightings western blot.

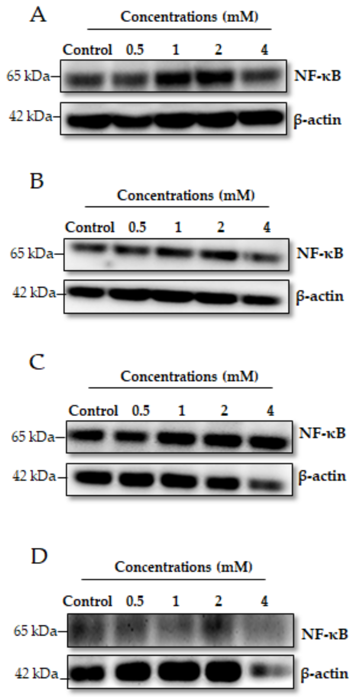

E

Concentrations	Densitometric analysis of relative NF-κB expression level			
Control	1.00	1.00	1.00	1.00
0.5 mM	0.73	0.90	0.89	1.11
1 mM	0.55	1.20	0.97	1.43
2 mM	0.74	1.44	1.10	1.62
4 mM	1.18	2.09	1.11	1.02

F

Descriptives of densitometric analysis of relative NF-κB expression level

Concentrations	N	Mean	Std. Deviation	Std. Error	95% Confidence Interval for Mean		Minimum	Maximum
					Lower Bound	Upper Bound		
Control	4.00	1.00	0.00	0.00	1.00	1.00	1.00	1.00
0.5 mM	4.00	0.91	0.16	0.08	0.66	1.16	0.73	1.11
1 mM	4.00	1.04	0.38	0.19	0.44	1.64	0.55	1.43
2 mM	4.00	1.23	0.39	0.20	0.60	1.85	0.74	1.62
4 mM	4.00	1.35	0.50	0.25	0.55	2.14	1.02	2.09

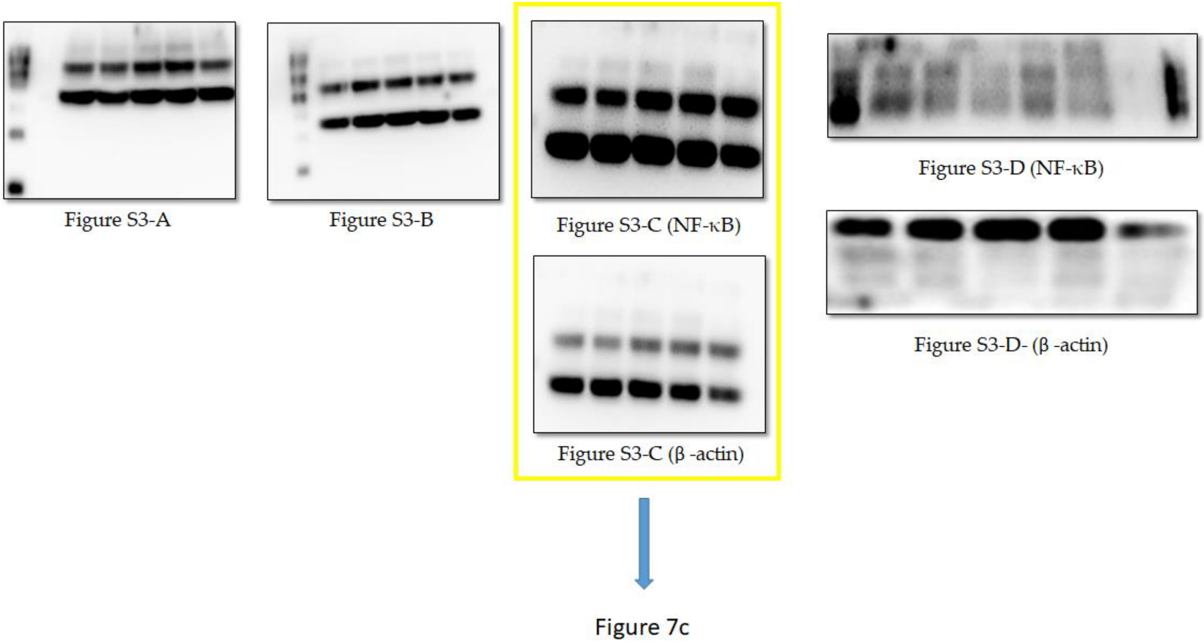

Figure S3. Whole western blot images of NF-κB (A-D) protein bands, densitometric analysis of relative NF-κB protein expression levels (E) and Descriptives of densitometric analaysis of relative NF-κB protein expression (F).

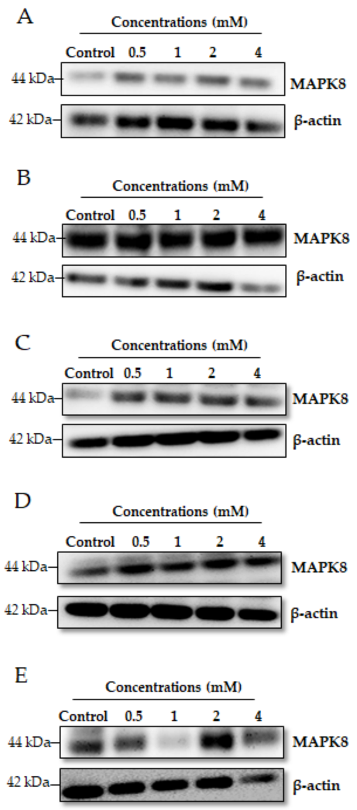

F

Concentrations	Densitometric analysis of relative MAPK8 expression level				
Control	1.00	1.00	1.00	1.00	1.00
0.5 mM	1.51	1.07	1.46	1.56	0.92
1 mM	1.14	0.80	1.56	1.26	0.25
2 mM	1.65	0.72	1.71	1.72	1.67
4 mM	2.06	1.36	1.81	2.07	1.88

G

Descriptives of densitometric analysis of relative MAPK8 expression level

Concentrations	N	Mean	Std. Deviation	Std. Error	95% Confidence Interval for Mean		Minimum	Maximum
					Lower Bound	Upper Bound		
Control	5	1.00	0.00	0.00	1.00	1.00	1.00	1.00
0.5 mM	5	1.30	0.29	0.13	0.95	1.66	0.92	1.56
1 mM	5	1.00	0.50	0.22	0.38	1.62	0.25	1.56
2 mM	5	1.49	0.44	0.19	0.95	2.03	0.72	1.72
4 mM	5	1.84	0.29	0.13	1.48	2.20	1.36	2.07

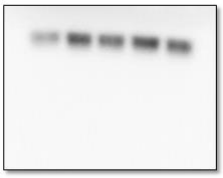
Figure S4-A (MAPK8)

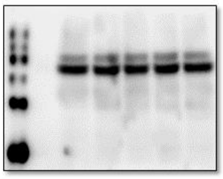
Figure S4-B (MAPK8)

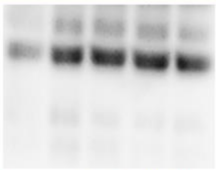
Figure S4-C (MAPK8)

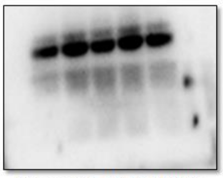
Figure S4-D (MAPK8)

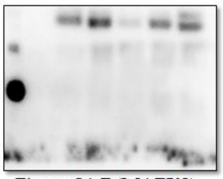
Figure S4-E (MAPK8)

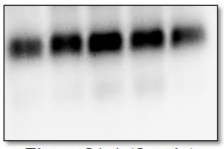
Figure S4-A (β-actin)

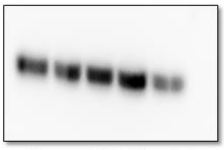
Figure S4-B (β-actin)

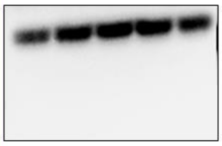
Figure S4-C (β -actin)

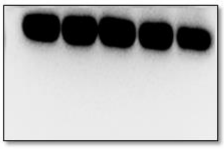
Figure S4-D (β-actin)

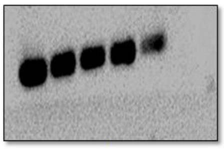
Figure S4-E (β -actin)

Figure 7e

Figure S4 Whole western blot images of MAPK8 (A-E) protein bands, densitometric analysis of relative MAPK8 protein expression levels (F) and descriptives of densitometric analysis of relative MAPK8 protein expression (G).
